# Ferromagnetic Cobalt Oxide With Structural Distortion and Oxidation State Changes for Hydrogen Sulfide Gas Detection

**DOI:** 10.1002/smll.73475

**Published:** 2026-04-21

**Authors:** Shin Joon Kang, Chang Yoon Kim, Min Chan Kim, Sunhyeong Kwon, Joon‐Shik Park, Hyung Mo Jeong

**Affiliations:** ^1^ School of Mechanical Engineering and Department of Smart Fab. Technology Sungkyunkwan University Suwon Gyeonggi‐do Republic of Korea; ^2^ Smart Sensor Research Center Korea Electronics Technology Institute (KETI) Seongnam Gyeonggi‐do Republic of Korea; ^3^ Department of Chemical Engineering Jeju National University Jeju Republic of Korea

**Keywords:** aligned structure, Co_3_O_4_, ferromagnetic, H_2_S gas sensor, lithiation‐based galvanostatic reduction

## Abstract

Fabricating ordered micropillar arrays via magnetic assembly of ferromagnetic materials can enhance H_2_S gas sensors. However, this approach is limited to iron oxide‐based ferromagnetic materials, which are unsuitable for H_2_S gas detection. Cobalt oxide is advantageous for H_2_S detection despite being intrinsically antiferromagnetic. The key challenge is thus inducing a net ferromagnetic moment in Co_3_O_4_ for the fabrication of micropillar arrays, designed to enhance H_2_S gas sensitivity. Herein, a lithiation‐based galvanostatic reduction (LiGr) process synthesizes a material (LiGr‐Co) featuring a Co_3_O_4_/CoO heterointerface, few‐nanometer‐scaled particles, and oxygen vacancies. The sub‐nanometer‐sized particles and oxygen vacancies significantly enhance H_2_S gas‐sensing performance by promoting the formation of short‐range order between Co^2+^ and Co^3+^ ions. Furthermore, the Co_3_O_4_/CoO heterointerface provides the pathway to induce the desired magnetism, as a resulting crystallographic mistilt at the interface generates the required magnetic moment. The aligned LiGr‐Co (A‐LiGr‐Co) sensor, under a magnetic field during the spray coating process, showed improved efficiency in gas adsorption and electron transfer, leading to a reduction of the base resistance and an enhancement of gas‐sensing capabilities. A‐LiGr‐Co demonstrated a high response (*R*_g_/*R*_a_) of 39.7 at 5 ppm and high selectivity for H_2_S detection at 150°C.

## Graphical abstract

High H_2_S gas sensitivity was improved by electrochemically engineering Co_3_O_4_/CoO heterostructures with oxygen vacancies and aligning nanosized particles via magnetic‐field‐assisted deposition. This alignment enhanced gas diffusion and electron transport, reducing base resistance. The resulting sensor exhibited high selectivity and a strong response (*R*_g_/*R*_a_) of 39.7 at 5 ppm H_2_S gas at 150°C.

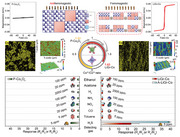

## 1 Introduction

The severe toxicity of hydrogen sulfide (H_2_S) gas at low parts‐per‐million (ppm) concentrations, with occupational safety limits mandated at 10 ppm, presents a critical challenge for public health and environmental monitoring [1, 2, 3, 4, 5, 6, 7, 8]. In this regard, chemiresistive gas sensors have been widely explored for H_2_S gas detection because of their simplicity, rapid response, and miniaturization potential. In particular, metal‐oxide semiconductors (MOSs) offer tunable physicochemical properties and structural versatility, making them promising candidates for gas‐sensing applications [9, 10]. The performance of MOS‐based gas sensors depends on both intrinsic material properties and extrinsic factors. Among these, the device architecture is critical as it determines gas diffusion and accessibility to active sites. Consequently, fabricating highly ordered, array architecture is a key strategy for enhancing this extrinsic performance.

A straightforward approach for fabricating ordered, micropillar arrays of sensing materials is to utilize magnetic‐field‐assisted assembly, which relies on ferromagnetic materials such as maghemite (γ‐Fe_2_O_3_) and magnetite (Fe_3_O_4_) [11, 12]. These oxides are primarily known for their selectivity toward gases like NO_2_ gas. Furthermore, their optimal sensing performance is typically realized only through hybridization with carbon‐based materials, which is essential to enhance their electrical conductivity [13]. In other words, the development of a multifunctional metal oxide material that synergistically combines both robust magnetism and high H_2_S gas selectivity remains a significant challenge.

Among these, Co_3_O_4_, a p‐type transition‐metal oxide, shows significant potential for H_2_S gas detection, owing to tunable structures and reactive Co^2+^ sites that enhance selectivity [14, 15, 16]. Generally, p‐type MOS gas sensors have a low baseline resistance, making them compatible for interfacing with electronic circuits, and they show small deviations in repeated measurements and are advantageous in terms of long‐term stability. However, its practical application is hindered by several challenges, including high operating temperatures (250°C–325°C) [17], poor gas sensitivity such as a low response and high limit of detection (LOD) [18, 19], and an inherent antiferromagnetic nature that limits advanced fabrication techniques.

The antiferromagnetism of pristine Co_3_O_4_ is determined by a long‐range super exchange pathway mediated by diamagnetic Co^3+^ ions [20]. To induce ferromagnetism, this transition requires a strategic increase in the concentration of magnetically active Co^2+^ cations [21]. Furthermore, short‐range magnetic correlation in the inverse spinel structure is induced not only by the Co^2+^/Co^3+^ distribution, but also by various internal parameters including particle sizes, surface defects, and distortions in the crystal structure [20, 22]. The formation of an interface in the Co_3_O_4_/CoO system can induce a net ferromagnetic moment. This phenomenon originates from a slight crystallographic mistilt between the two cubic structures at the interface, which prevents the perfect compensation of antiferromagnetic spins [23]. In other words, controlled partial reduction of Co_3_O_4_ nanoparticles (NPs) is a facile yet powerful method to generate these uncompensated spins by forming such an interface. This ability to provide robust magnetic properties onto Co_3_O_4_ materials significantly expands their potential for applications in magnetic‐field‐induced aligned micropillar H_2_S gas sensors.

We investigated a facile method to control the heterostructure of cobalt oxide NPs with ferromagnetic properties using the lithiation‐based galvanostatic reduction (LiGr) method to form aligned micropillar arrays on a sensor device. These ferromagnetic properties of cobalt oxide, combined with the micropillar arrays, enabled the formation of tight interfacial contacts for efficient electron transfer. Additionally, heterostructured Co_3_O_4_ surfaces with partially reduced CoO provide favorable interfaces that serve as effective adsorption sites for H_2_S gas molecules. To convert the pristine Co_3_O_4_ (P‐Co_3_O_4_) properties from antiferromagnetic to ferromagnetic, the fragmentation of Co_3_O_4_ particles into nanoscale domains facilitates the formation of short‐range electron transport pathways through oxygen vacancies and local ordering between Co^3^^+^ and Co^2^^+^ ions, thereby inducing magnetic properties via the LiGr method. High‐resolution transmission electron microscopy (HR‐TEM) and X‐ray diffraction (XRD) analyses, along with density functional theory (DFT) calculations on the specific facets of Co_3_O_4_ partially reduced to CoO, were employed to systematically characterize the Co_3_O_4_‐based material synthesized using the LiGr method, elucidating its high selectivity toward H_2_S gas molecules. Antiferromagnetic Co_3_O_4_ is converted to ferromagnetic material via the lithiation‐based galvanostatic reduction method (LiGr‐Co), which enabled the facile formation of electrode channels through a micropillar array under a magnetic field, significantly improving the H_2_S gas‐sensitivity by considerably reducing the base resistance.

## 2 Results and Discussion

### 2.1 Generation and Control of Magnetic and Microstructural Properties of Cobalt Oxide Nanoparticles by LiGr Process

**FIGURE 1:**
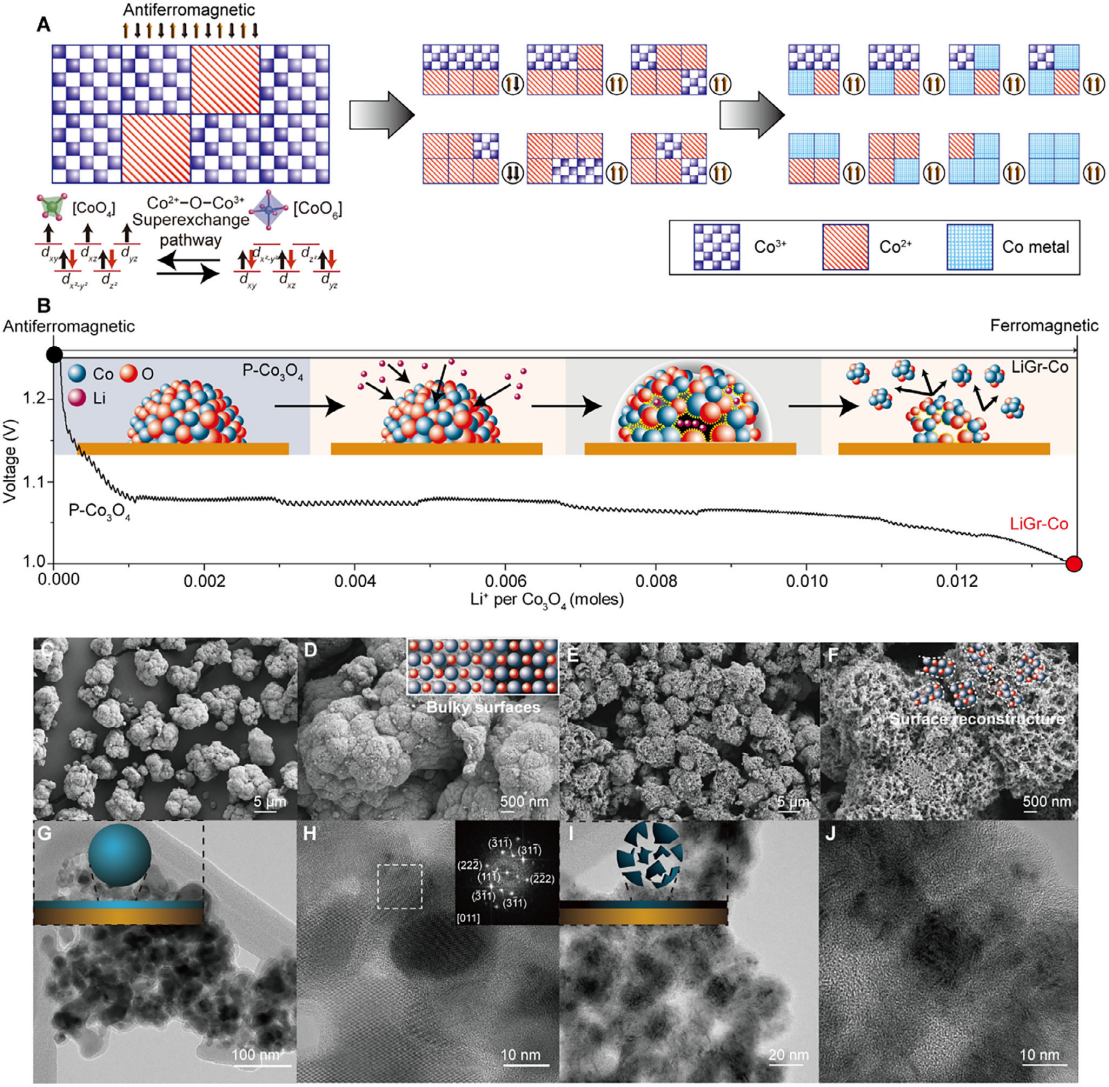
(A) Illustration of the structural evolution of Co_3_O_4_ particles with the supporting effect of cations on the atomic structure of Co_3_O_4_ during the LiGr processes. The crystalline unit cell of pristine Co_3_O_4_ includes the local structures and the related crystal field diagram for high‐spin (HS) cobalt atoms in tetrahedral coordination and low‐spin (LS) cobalt atoms in octahedral coordination. The black and gray arrows show the up and down spin orientations according to the experimental antiferromagnetic ordering, respectively. (B) Galvanostatic reduction profile and schematic illustration of the fabrication of ferromagnetic cobalt oxide (LiGr‐Co) via the LiGr process. SEM images of (C,D) P‐Co_3_O_4_ and (E,F) LiGr‐Co at low and high magnifications. TEM images of (G,H) P‐Co_3_O_4_ and (I,J) LiGr‐Co at low and high magnifications. The inset in (H) is the corresponding SAED pattern.

The crystal structure of Co_3_O_4_, which is a normal spinel with Fd‐3m symmetry, features a cubic, close‐packed arrangement of oxygen anions. Within this structure, one‐eighth of the tetrahedral sites are filled by high‐spin Co^2+^ cations, and half of the octahedral sites are occupied by low‐spin Co^3+^ cations. Each Co^2+^ cation ($e_{g}^{4}t_{2g}^{3})$ was encircled by its four nearest neighbors with opposite spins [21, 24]. In contrast, Co^3+^ cations have a closed‐shell configuration ($t_{2g}^{6}$) and exhibit no magnetic moment, as shown in Figure 1A. Furthermore, the overall magnetism arises owing to the extended super exchange pathway of Co^2+^─O─Co^3+^─O─Co^2+^, with Co^3+^ ions positioned at the intermediate sites between O^2−^ ions. In P‐Co_3_O_4_, the Co^3+^ ions in the octahedral sites have almost no spin, causing the Co^2+^ ions to align in an antiparallel manner. In this case, the bond angle of the super exchange pathway was 180°, resulting in an antiferromagnetic behavior [20]. We explored the LiGr process for ferromagnetic cobalt oxide materials with particle size changes and numerous defects by modifying antiferromagnetic P‐Co_3_O_4_ NPs [25, 26]. Initially, P‐Co_3_O_4_ is partially reduced electrochemically by Li^+^ ions, which act as structure‐transforming chemical agents. The LiGr pathway involves a Li‐intercalation process followed by conversion to Li_x_Co_3_O_4_ [27]; then, after washing, LiGr‐Co powder is obtained. We also confirmed the absence of the Li ion influence on the controlled materials through the X‐ray photoelectron spectroscopy (XPS) spectra of cobalt oxide following the LiGr process, indicating that the Li species were eliminated after a simple washing step (Figure S1). Disintegration, the formation of a few nanometer‐scaled cobalt oxide particles with ferromagnetic properties owing to the expansion of phase separation during the LiGr process, depends on the appropriate extent of Li‐ion insertion per Co_3_O_4_ structure (Figure 1B).

The structural changes in P‐Co_3_O_4_ caused by different degrees of LiGr, based on the applied discharge potentials (1 V vs. Li/Li^+^), were analyzed using scanning electron microscopy (SEM) (Figure 1C–F). Initially, the SEM images of P‐Co_3_O_4_ show significant agglomeration with a bulky surface and a lack of distinct shapes (Figure 1C,D). On the other hand, the well‐dispersed porous nanostructure of LiGr‐Co is visible in a portion of the SEM images after the LiGr process with Li^+^ ions (Figure 1E,F). The structures of P‐Co_3_O_4_ and LiGr‐Co were further investigated using transmission electron microscopy (TEM) (Figure 1G–J). P‐Co_3_O_4_ exhibited smooth surfaces with no notable defects, and the selected area electron diffraction (SAED) patterns indicated the presence of several dot patterns, confirming the single‐crystal nature of the as‐prepared P‐Co_3_O_4_ (Figure 1G,H). However, after the LiGr process with Li^+^ ions, a reduction in the grain size of the single‐crystalline cobalt oxide was observed (Figure 1I,J). These cracks and grain boundaries expanded and propagated at 1 V in LiGr‐Co. To further investigate the structural changes induced by the LiGr process, the particle size distributions of the samples were quantitatively evaluated using ImageJ software. As shown in Figure S2A–D, nearly 80 particles were randomly selected from various TEM images to estimate the mean particle size (Figure S2A,C). The calculated mean particle size of the pristine P‐Co_3_O_4_ NPs was 14.82 ± 1.11 nm (Figure S2B). Remarkably, after the LiGr reduction process, the mean particle size of the LiGr‐Co significantly decreased to 4.87 ± 0.26 nm (Figure S2D). This statistical distribution clearly demonstrates that the LiGr process effectively reduces the grain size of the single‐crystalline cobalt oxide, which is highly beneficial for providing abundant active sites for gas sensing reactions. A controlled structural design to reduce the grain size of Co_3_O_4_ is expected to leverage the finite‐size effect toward short‐range magnetic states [28, 29]. The intercalation of Li atoms into the Co_3_O_4_ crystal, as shown in the TEM images, indicated the formation of numerous oxygen vacancies and other defects.

**FIGURE 2:**
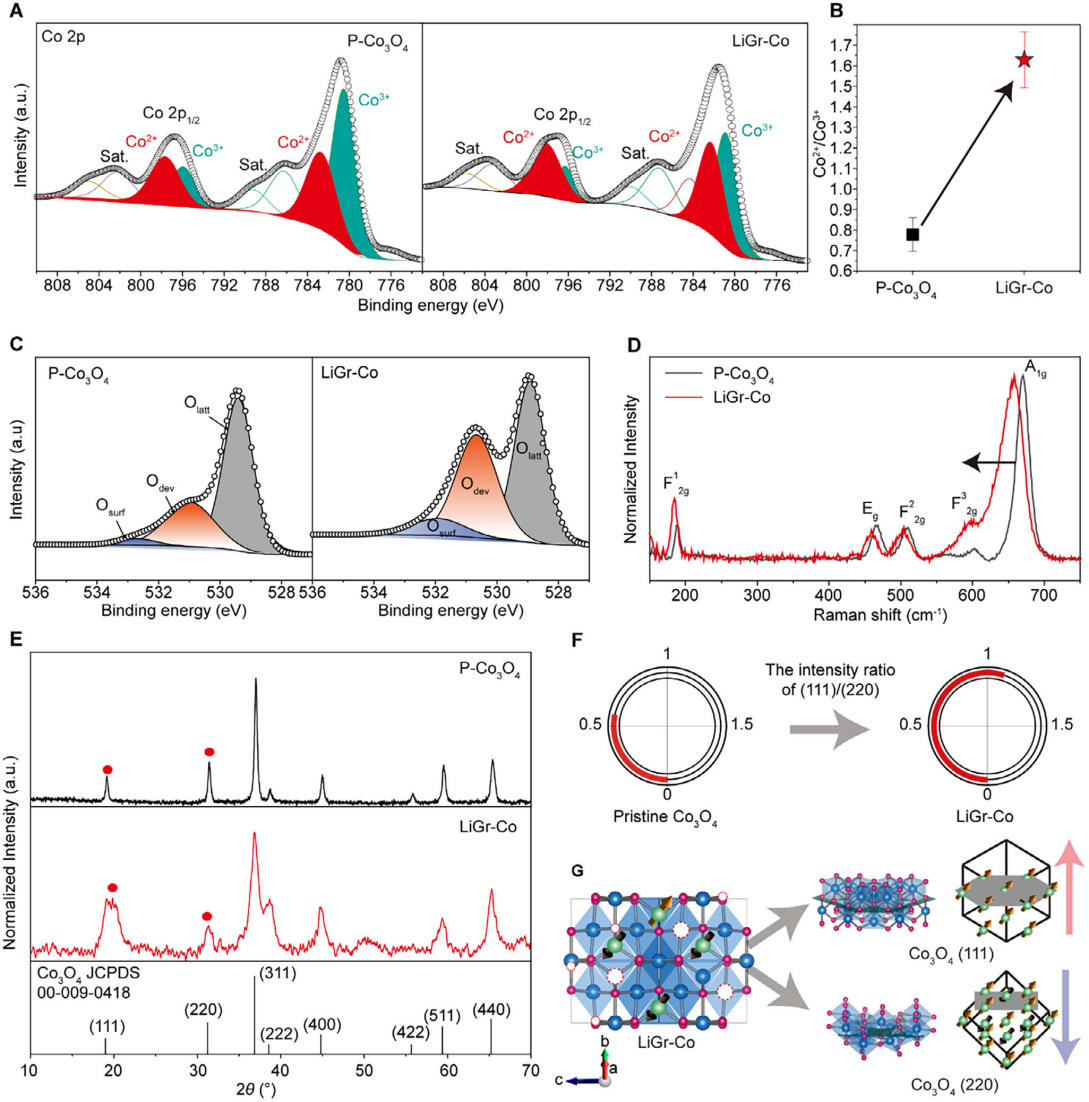
(A) high‐resolution XPS spectra of Co 2p for P‐Co_3_O_4_ and LiGr‐Co, and (C) high‐resolution XPS spectra of O 1s for P‐Co_3_O_4_ and LiGr‐Co. (D) Ex situ Raman spectra of P‐Co_3_O_4_ and LiGr‐Co. (E) XRD patterns of P‐Co_3_O_4_ and LiGr‐Co. The bottom vertical lines represent the standard PDF card of Co_3_O_4_. (F) Relative XRD intensity ratio along (111) and (220). (G) Schematic of the crystal structure of Co_3_O_4_ (111) and (220) planes.

Then, structural evolution enhances short‐range ferromagnetic coupling, which is facilitated by the super exchange interactions mediated by oxygen between the Co^2+–^Co^2+^ or Co^2+–^Co^3+^ pairs. In addition, the LiGr process leads to oxygen vacancies in the Co–O system, thereby altering the local electronic environment by introducing unpaired d electrons. These ions were inserted into the starting materials, leading to the formation of cobalt oxide with a large ratio of Co^2+^/Co^3+^ nanocomposites. The characteristic peaks, verified by the deconvolution of the Co 2p spectra, are presented in Figure 2A. The two spin doublets are resolved into four peaks at approximately 778.9 eV (Co 2p^3/2^), 794.3 eV (Co 2p^1/2^), 780.3 eV (Co 2p^3/2^), and 796.1 eV (Co 2p^1/2^), with additional satellite peaks [30, 31]. The peaks near 778.9 and 794.3 eV indicate octahedral Co^3+^ in Co_3_O_4_, whereas the peaks around 780.3 and 796.1 eV correspond to tetrahedral Co^2+^. As shown in Figure 2B, the Co^2+^/Co^3+^ ratio for LiGr‐Co (1.63) was approximately twice as large as that for P‐Co_3_O_4_ (0.84). Typically, a larger Co^2+^/Co^3+^ intensity ratio suggests an increased number of surface oxygen defects, which enhance gas adsorption, thus facilitating H_2_S gas molecule adsorption and electron transfer [32, 33]. Furthermore, defect generation and structural evolution can be controlled by the applied discharge potential, as shown in Figure 1B.

Figure 2C shows peaks at 529.8, 531.2, and 532.5 eV, corresponding to lattice oxygen with full complementation, oxygen‐deficient sites, and hydroxyl species from the surface‐adsorbed water molecules, respectively. The notable increase in the peak intensity at 531.2 eV for LiGr‐Co indicated a high concentration of oxygen vacancies [34]. The LiGr method of cobalt oxide crystals reduces the coordination of the Co─O bond, leading to numerous oxygen vacancies. These vacancies serve as sites for gas adsorption and significantly enhance the electron transfer efficiency of reactions with the target gas [35]. The Raman spectrum of the Co_3_O_4_ NPs displayed three distinct peaks at approximately 468, 510, and 670 cm^−1^, corresponding to three Raman‐active modes (E_g_, F_2g_, and A_1g_) (Figure 2D) [31]. These modes involve the redistribution of Co^2+^ and Co^3+^ cations and combined vibrations of the tetrahedral and octahedral sites. This comparison confirms the pure spinel structure of the sample before and after the LiGr process. Additionally, compared with P‐Co_3_O_4_, the peak positions of these active modes shift to lower wavenumbers by approximately 10–20 cm^−1^, likely owing to a reduction in grain size. These shifts are attributed to the phonon confinement effect, which typically depends on the shape and size of the NPs [36, 37]. Figure 2E shows the XRD patterns of the cobalt oxide NPs before and after the addition of LiGr. Diffraction peaks were observed at approximately 2*θ* = 31°, 36.5°, 44.3°, 59.2°, and 65°, corresponding to the (220), (311), (400), (511), and (440) reflections, respectively, of the cubic spinel structure of Co_3_O_4_ (JCPDS reference code No. 00‐009‐0418). All the prominent reflections in this pattern can be attributed to the Co_3_O_4_ phase. A broad peak observed at a 2*θ* range between 10° and 70° can be interpreted as part of the Co_3_O_4_ phase. This significant broadening indicates the presence of small crystalline domains within this phase. We inferred that the NPs consisted of Co_3_O_4_ with very small crystalline domains. Additionally, as Li^+^ ions were inserted into the lattice of the Co_3_O_4_ structure, the intensity of the peak corresponding to the (220) plane decreased owing to structural strain, whereas the intensity of the (111) plane increased [38, 39]. Consequently, as shown in Figure 2F, the intensity ratio of (111)/(220) significantly increased from 0.53 to 1.23. Extensive analyses revealed that particles with small crystalline domains and high oxygen vacancies exhibited a notable variation in the intensity ratio of the (111)/(220) XRD diffraction peaks (Figure 2G), a structure originating from the reduced crystalline size that is considered favorable for gas adsorption behavior. Furthermore, the (111) plane contains only Co^2+^ cations, whereas the {110} family of planes is predominantly composed of Co^3+^ cations [38]. The atomic structure of the (111) plane promotes magnetic coupling between Co ions, leading to strong ferromagnetism, with its high‐density Co_3_O_4_ (111) plane with Co and O active sites enhancing the adsorption and catalytic reactions of H_2_S gas molecules [40]. These active sites drive redox reactions, resulting in a strong gas‐sensing response. Surface differential diffraction studies confirmed that the Co^2+^ cations were exclusively present in the (111) plane. Therefore, for our LiGr‐Co samples, it is highly likely that Co^2+^ cations on the exposed surface planes in the Co_3_O_4_ nanostructures are formed owing to surface oxygen deficiency.

### 2.2 Gas Sensor Device Fabrication Using Magnetic Properties of Cobalt Oxides

**FIGURE 3:**
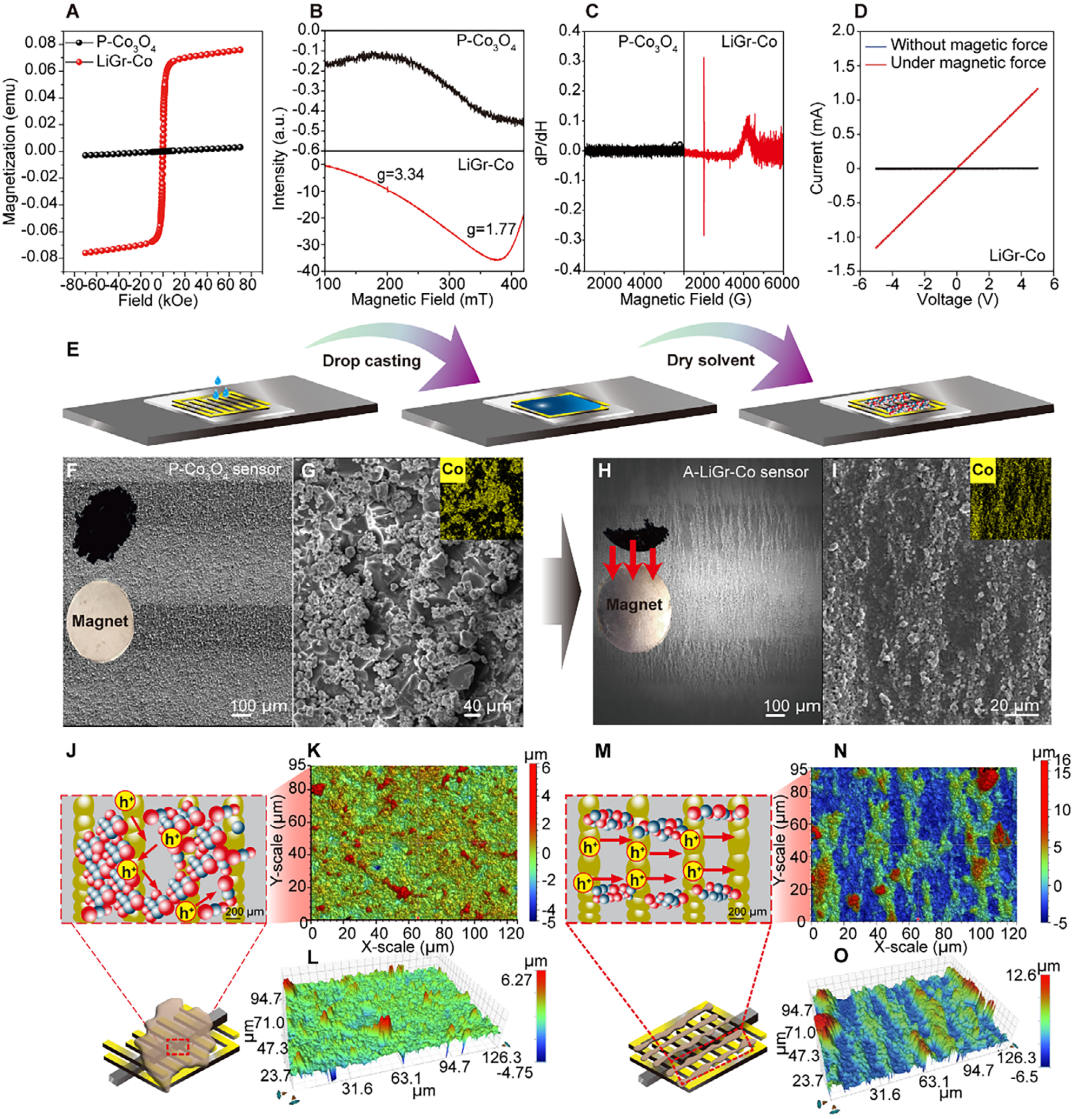
(A) M–H hysteresis curve recorded at room temperature for the P‐Co_3_O_4_ and LiGr‐Co samples. (B) ESR spectra of P‐Co_3_O_4_ and LiGr‐Co NPs, (C) differential ESR spectra (dP/dH) versus the magnetic field. (D) *I–V* curves of the LiGr‐Co sensing devices without/with magnetic‐field‐induced alignment. (E) Schematic of depositing gas‐sensing materials with drop‐coating. SEM images of (F,G) coated P‐Co_3_O_4_ film and (H,I) A‐LiGr‐Co film on the sensing device in low and high magnifications. The insets in (F and H) and (G,I) are the corresponding optical images showing P‐Co_3_O_4_ and A‐LiGr‐Co powder on a substrate with a magnet placed beneath and the EDS mapping of Co element, respectively. The Co_3_O_4_ powder appears as a dark spot. Schematic depicting the molecular arrangement and transport of holes (*h* ^+^) in the (J) P‐Co_3_O_4_ film and (M) A‐LiGr‐Co. (K,N) Surface topography map obtained from 3D profiler for P‐Co_3_O_4_ and A‐LiGr‐Co, showing the microstructural features, and 3D surface profile of the (L) P‐Co_3_O_4_ and (O) A‐LiGr‐Co film obtained from 3D profiler, providing a detailed view of the surface topography. The color scale indicates the height variations in micrometers.

By controlling the size and valence state of the Co_3_O_4_ NPs, we can investigate the influence of the exposed surfaces on the magnetic properties, which exhibit different Co^2+^/Co^3+^ ratios through the resulting surface terminations, as shown in Figure 3A–D. To study the magnetic properties of the samples, we measured the P‐Co_3_O_4_ and LiGr‐Co hysteresis loops at room temperature. The magnetization plots as a function of the applied magnetic field (H) are shown in Figure 3A. This reveals that the P‐Co_3_O_4_ has a nonmagnetic response, as expected. Meanwhile, the room‐temperature ferromagnetic behavior of the LiGr‐Co NPs exhibited significant intensity with a saturation magnetization at 10 kOe of 54.4 emu. The magnetic properties of LiGr‐Co NPs can be ascribed to several factors related to the presence of unpaired electron spins. These factors include (i) uncompensated Co^2+^ ions on the surface, (ii) the nanoscale size of the particles, and (iii) surface oxygen vacancies or particle interfaces [41]. In addition, electron spin resonance (ESR) was conducted to gain further insight into the magnetic interactions and spin correlations at a microwave frequency of 9.38 GHz (Figure 3B,C). The *g‐*value can be determined from the operating frequency and the resonance magnetic field based on the following Equation (1) [42].

(1) $$g=\frac{h\nu }{\beta B_{0}}\;$$

In this formula, *h* represents the Planck constant (6.626  ×  10^−34^ J s), ν defined for the measurement frequency (9.5 GHz), β denotes the Bohr magneton (9.274  ×  10^−28^ J G^−1^), and *B*_0_ indicates the applied magnetic field at resonance. Among the Co ions, diamagnetic Co^3+^ ions from Co_3_O_4_, which have a 3*d* ^6^ configuration, do not appear in the ESR studies [43, 44]. In contrast, ESR focuses on active Co^2+^ ions with a 3*d* ^7^ configuration. The ESR spectra of tetrahedrally coordinated Co^2+^ ions typically display a prominent sharp peak and a secondary, broader, and weaker peak. Based on the ESR spectrum, the LiGr‐Co NPs exhibited a stronger ESR signal with the highest peak intensity compared with the P‐Co_3_O_4_ NPs, which has also been observed in antiferromagnetic NPs within the superparamagnetic regime, as reported in previous studies [45]. This suggests an increase in the number of single electrons in the LiGr‐Co NPs. In addition, the LiGr‐Co NPs have higher concentrations of oxygen vacancies and Co^2+^ ions. The primary factor influencing the determination of the ESR resonance parameters was the magnetic dipole interactions among the Co_3_O_4_ NPs. While the true g‐factor of a free electron is very close to 2, the *g*‐value for Co^2+^ ions is known to be highly sensitive to the distortion of the octahedral environment, theoretically allowing it to range anywhere between 2 and 9 [46]. For both P‐Co_3_O_4_ and LiGr‐Co NPs, these interactions were nearly identical, as indicated by the similar broad peaks around 370 mT. Unlike P‐ Co_3_O_4_, LiGr‐Co showed an evident axial‐type ESR signal for Co^2+^ spins centered at *g* = 3.34, indicating strong spin‐orbit coupling caused by a severely distorted surface lattice. This specific distortion creates a dense population of uncompensated surface spins, which act as the fundamental origin of the observed ferromagnetic interactions [47, 48]. Figure 3C shows the ESR derivative spectra, with the derivative curves detected in the measurement field *H* < 2000 G, identified as the ferromagnetic signal. Even after depositing LiGr‐Co (2 mg mL^−1^) on the surface of the electrodes via standard spray coating, with and without magnetic‐field‐induced alignment, Figure 3D shows typical current–voltage (*I–V*) curves for the two gas‐sensing devices in air. The multichannel device with the aligned spheres showed a higher base current than the device with a random arrangement. This indicates that the LiGr‐Co particles were more tightly assembled, creating an effective conducting channel under the influence of the magnetic field. The diluted P‐Co_3_O_4_ and LiGr‐Co dispersions were sequentially spray‐coated onto an alumina substrate with an interdigitated Au electrode to create an aligned Co_3_O_4_ structure, as shown in Figure 3E (Further details regarding the device fabrication process can be found in the Experimental Section).

The morphological analysis of the Co_3_O_4_ NPs revealed significant differences between the pristine and magnetically aligned samples. Initially, the P‐Co_3_O_4_ NP powder was examined using optical microscopy (Figure 3F, inset), which showed a random distribution of the particles on the substrate with the magnet positioned nearby. Furthermore, the low‐ and high‐resolution SEM images of P‐Co_3_O_4_ provided a detailed nanostructure of the sensor device (Figure 3F,G), indicating an irregular and loosely packed arrangement of NPs, exhibiting antiferromagnetic properties. The SEM‐energy‐dispersive X‐ray spectroscopy (EDS) mapping of Co, O, and Au revealed a random distribution and disconnected morphology of the Co_3_O_4_ particles (Figure 3G inset and Figure S3). In contrast, upon subjecting the cobalt oxide powder to a magnetic field of 0.8 T during the spray coating process, a pronounced alignment was observed (Figure 3H), where the particles aligned themselves in response to the magnetic field lines, as depicted by the directional arrows toward the magnet. LiGr‐Co particles with the same concentration (2 mg mL^−1^) were aligned perpendicular to the interdigital electrodes, demonstrating that the Co_3_O_4_ particles were influenced by the magnetic field, as depicted in the inset of Figure 3H. This magnetic alignment was further corroborated by the SEM image of the magnetically aligned LiGr‐Co (A‐LiGr‐Co) (Figure 3I), which showed a more organized and densely aligned nanostructure with particles forming chain‐like structures aligned in the direction of the magnetic field. Moreover, SEM‐EDS mapping confirmed the continuous presence of Co and O without interruption (Figure 3I inset, S4). Figure 3J–O illustrates the surface morphology and structural configuration of the cobalt oxide‐based sensing material, depicting the 2D and 3D surface profiles obtained using a profilometer, and highlighting the intricate texture and variation in the surface roughness. In the P‐Co_3_O_4_‐based sensor device, the randomly distributed Co_3_O_4_ NPs, which are majority hole carriers, follow a complex path through the conducting channel between the two electrodes (Figure 3J). The surface topography was analyzed using height maps (Figure 3K,N) and 3D surface plots (Figure 3L,O), where the pristine sample displayed a relatively uneven surface with scattered height variations. The magnetically aligned A‐LiGr‐Co sensor exhibited a distinct and periodic surface pattern, indicating a more ordered arrangement. This structural transformation owing to magnetic alignment suggests that the application of a magnetic field can significantly influence the packing density and orientation of LiGr‐Co NPs, and the tight contact between LiGr‐Co NPs facilitates more efficient transport of charged carriers (Figure 3M). Furthermore, the process resulted in average thicknesses of approximately 11 and 19.1 µm, respectively. Note that, by regulating the magnetic field and concentration of the particle suspension, a conducting channel composed of multilayer particles can be achieved. The significant reduction in the base resistance (Figure S5), indicates a marked improvement in the effectiveness of the material as a gas sensor. Specifically, the lower base resistance of the LiGr‐Co and A‐LiGr‐Co samples, compared with that of P‐Co_3_O_4_, suggests enhanced sensitivity and a lower operating temperature for detecting gas molecules. This enhancement can be attributed to the improved conductivity and greater surface area available for gas interactions. The magnetic‐field‐induced alignment allowed us to produce fewer Co_3_O_4_ particles, maximizing the surface area available for gas molecule detection.

### 2.3 H_2_S Gas‐Sensing Performance of Magnetically Aligned Co_3_O_4_ Micropillar Arrays

**FIGURE 4:**
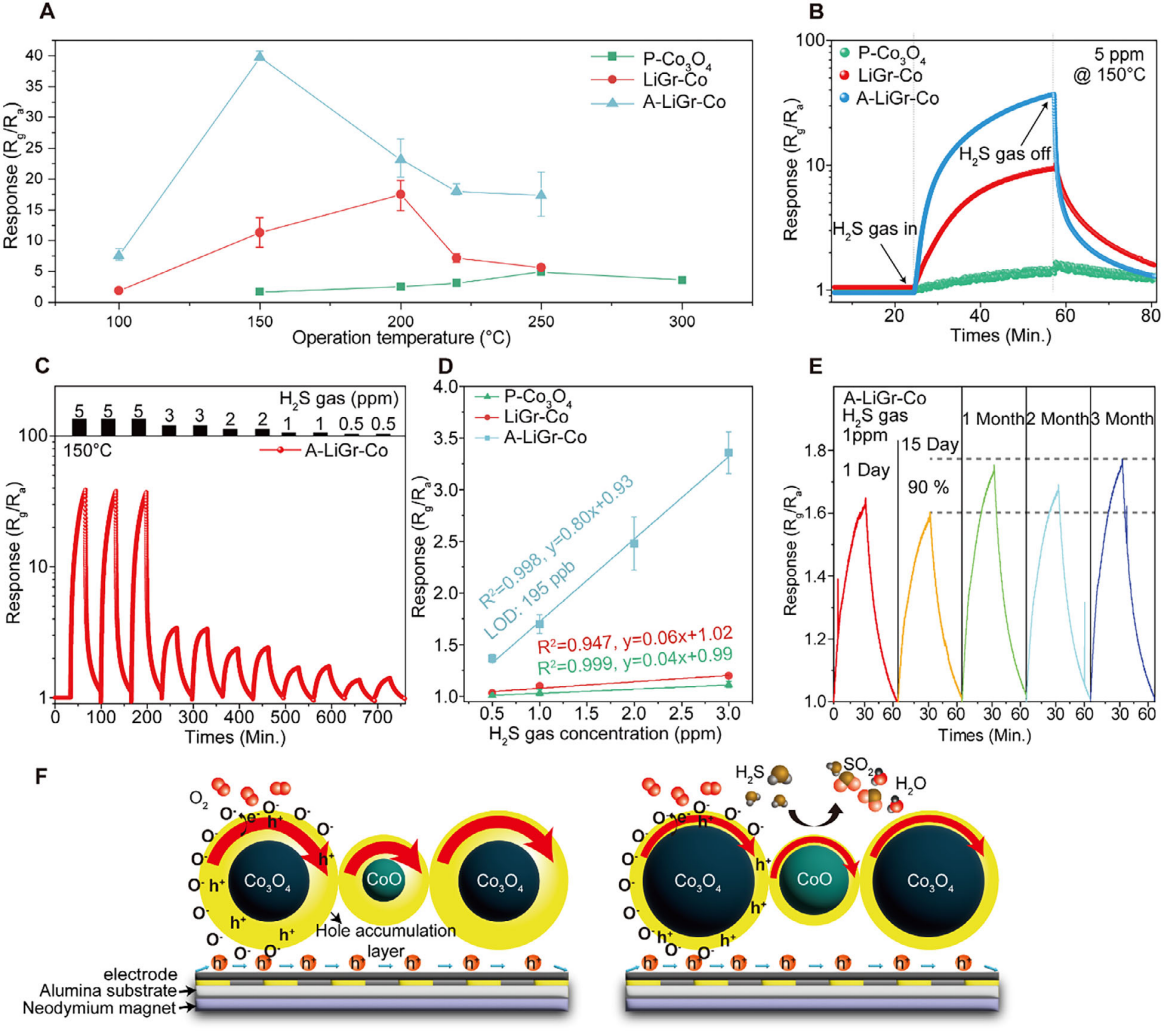
(A) Responses of the P‐Co_3_O_4_, LiGr‐Co, and A‐LiGr‐Co based gas sensors toward 5 ppm H_2_S gas at different temperatures ranging from 100°C to 300°C. (B) Dynamic response and recovery behaviors of the P‐Co_3_O_4_, LiGr‐Co, and A‐LiGr‐Co based gas sensors exposed to 5 ppm H_2_S gas at their optimal temperatures of 150°C, 200°C, and 250°C, respectively. (C) Gas‐sensing performance of the A‐LiGr‐Co sensor at various H_2_S gas concentrations. (D) Dynamic response of the A‐LiGr‐Co sensor to different concentrations of H_2_S gas (from 5 to 0.1 ppm) at 150°C and the calibration curves for the P‐Co_3_O_4_, LiGr‐Co, and A‐LiGr‐Co sensors, depicting the relationship between the sensor response and the H_2_S gas concentration. (E) Long‐term stability test of the response of the A‐LiGr‐Co sensor to 20 ppm H_2_S gas over 3 months at 150°C. (F) Schematic of the gas‐sensing mechanism of A‐LiGr‐Co in the presence of H_2_S gas.

A gas sensor test system was employed to evaluate the gas‐sensing performance of the fabricated sensor, as shown in Figure S6. The system is equipped with mass flow controller systems, multimeters, sealed test chambers, and precise gas flow control units. Various gases were introduced into the chamber through individual lines connected to gas cylinders, each regulated by a valve and mass flow controller to ensure accurate concentration control. The fabricated gas sensor chip was placed inside the sensor chamber and connected to the two‐probe measurement system via electrical probes to systematically evaluate the gas sensing performance. The electrical response of the sensor was monitored in real time using a computer interface that records the resistance changes as a function of time. Detailed information regarding the precise structure and dimensions of the sensor device is thoroughly described in the Experimental Section. This setup enabled precise and repeatable measurements of the resistance variation of the sensor upon exposure to different target gases. The gas‐sensing performance of the Co_3_O_4_‐based samples was evaluated using a bottom‐heated gas sensor in a static system, and the gas sensors were prepared by depositing equal volumes of solution (2.5 mL at a concentration of 2 mg mL^−1^) using a drop‐casting method. The P‐Co_3_O_4_, LiGr‐Co, and A‐LiGr‐Co‐based H_2_S gas sensors were tested with 5 ppm H_2_S gas at various operating temperatures. As the operating temperature affects electron mobility and material conductivity, it significantly influences the gas sensor response. Therefore, the optimal conditions for detection with a 5 ppm concentration of H_2_S were determined based on the response curves of P‐Co_3_O_4_, LiGr‐Co, and A‐LiGr‐Co NPs at temperatures ranging from 100°C to 300°C, as shown in Figure 4A. As the operating temperature increases, the thermal motion of the target gas molecules is enhanced, leading to improved diffusion toward the sensing surface and thus, an increase in sensitivity. However, further temperature elevation increases the thermal motion of the adsorbed species, resulting in a higher desorption rate, which in turn leads to a decrease in sensitivity [49]. For the A‐LiGr‐Co gas sensor, a response to H_2_S gas was observed even at relatively low temperatures, owing to its low baseline resistance. The optimal sensing temperature was identified as 150°C, at which a maximum response of 39.7 was shown. As the temperature increased further, the response gradually decreased to 23.1 at 200°C and 18 at 220°C. In comparison, the LiGr‐Co gas sensor showed a response of 11.3 at 150°C, which increased to 17.5 at 200°C but significantly decreased to 7.2 at 220°C, corresponding to a 60% decrease. On the other hand, the P‐Co_3_O_4_ gas sensor exhibited the highest response of 5 at 250°C. Figure 4B shows the gas response and recovery curves of these samples toward 5 ppm of H_2_S gas at 150°C. Notably, A‐LiGr‐Co exhibited the highest response among all the samples. This led to enhanced charge‐transfer properties within the cobalt oxide crystal and a reduction in particle size, which in turn reduced the degree of aggregation. The degree of aggregation of the cobalt oxide particles gradually decreased under the influence of a magnetic field, leading to more effective exposure to H_2_S molecules and thus an improvement in the gas‐sensing performance. Additionally, the LiGr process creates numerous oxygen vacancies in cobalt oxide, making it an effective gas‐sensing material.

An exposure time of 1000 s was defined as the effective response time for the effective evaluation of the sensing performance of the device. The gas response and recovery curves for different H_2_S gas concentrations, ranging from 5 to 0.5 ppm, are shown in Figure 4C. The A‐LiGr‐Co gas sensor exhibited a relatively superior response to H_2_S gas compared with the resistance variation and response curves of the P‐Co_3_O_4_ sensor under different H_2_S gas concentrations, as shown in Figure S7. In addition, the response value increased significantly with various H_2_S gas concentrations, with 1 ppm of H_2_S gas resulting in a response with a response time of ∼1411 s (Figure S8).

This phenomenon can be physically attributed to the massive quantity of gas molecules adsorbed onto the abundant active sites of the structurally optimized cobalt oxide NPs [59, 60]. Even at a low H_2_S gas concentration of 500 ppb, the device still exhibited a response value of up to 1.29, indicating excellent gas‐sensing performance of the magnetic‐field‐induced alignment multichannel device at 150°C. The relationship between the response value and the H_2_S concentration in Figure 4D shows that the gas response value is linearly proportional to the H_2_S concentration at higher concentrations, mainly because of the supersaturated absorption of H_2_S gas on the cobalt oxide NP surface. The LOD of the A‐LiGr‐Co‐based gas sensor device was calculated as 195 ppb. As summarized in Table 1, the proposed A‐LiGr‐Co sensor exhibits highly competitive sensing performance toward H_2_S gas compared with most recently reported Co_3_O_4_‐based sensors. The competitive sensing performance is primarily ascribed to the synergistic effects of the LiGr process‐derived cobalt oxide heterostructure and the well‐aligned structural configuration formed under a magnetic field. Figure 4E illustrates the long‐term stability of the device over three months. The device continues to demonstrate an excellent sensing performance with no degradation in gas‐sensing performance over time. Furthermore, the humidity stability of the LiGr‐Co sensor was tested at 150°C toward 3 ppm H_2_S gas under varying relative humidity (RH) levels ranging from dry to 80% RH, as shown in Figure S9. The corresponding responses were measured as 3.78, 2.01, 1.73, 1.61, and 1.61 at dry, 20%, 40%, 60%, and 80% RH, respectively. As the humidity level increases, the baseline resistance gradually increases. This is primarily attributed to the competitive adsorption of water molecules. The adsorption of water molecules alters the surface oxygen species and band bending, effectively reducing the hole concentration near the surface in the p‐type semiconductor, leading to an increase in the baseline resistance.

**TABLE 1:** Comparison of the H_2_S gas sensing performance of the A‐LiGr‐Co based gas sensor with other recently reported cobalt oxide‐based gas sensors.

Sensing Materials	Synthetic methods	Conc. (ppm)	Response (*R*_a_/*R*_g_ or *R*_g_/*R*_a_)	Temp. (°C)	Detection limit (ppm)	Refs.
Co_3_O_4_ nanorods	Hydrothermal method	100	4.57	225	10	[16]
Co_3_O_4_ tubes	Biomass template	100	50.2	170	0.1	[50]
Co_3_O_4_ nanoparticles‐decorated WO_3_ nanopolyhedra (Co_3_O_4_ NPs/WO_3_ NPHs)	Hydrothermal method	100	1220	200	0.1	[51]
Co_3_(HITP)_2_ with Co_3_O_4_ nanoparticles (Co_3_O_4_@Co_3_ (HITP)_2_)	Solvothermal method	10	3	25	0.13	[52]
In‐doped Co_3_O_4_ nanoflowers	Solvothermal method	50	19	180	1.8	[53]
Co_3_O_4_/WO_3_	Hydrothermal method	10	12.85	280	0.002	[54]
CuO/Co_3_O_4_	Hydrothermal and electrospinning	25	2.94	200	0.5	[55]
In_2_O_3_/Co_3_O_4_	MOF‐on‐MOF templated pyrolysis	100	110.2	100	0.323	[56]
Cr‐doped Co_3_O_4_	Chemical co‐precipitation	10	36.21	230	1.0	[57]
MOF‐derived Co_3_O_4_ Ns	Co‐precipitation method	100	18.03	250	0.5	[58]
A‐LiGr‐Co NPs	Electrochemical LiGr process	5	39.7	150	0.195	This work

### 2.4 Theoretical Study of H_2_S Gas Selectivity on the LiGr‐Co Sensing Material

Generally, Co_3_O_4_, which is a p‐type semiconductor, has a narrow bandgap (*E*_g _= 1.7 eV) and a work function of 5.2 eV [61, 62]. The LiGr process of Co_3_O_4_ led to the partial formation of CoO clusters on the surface of Co_3_O_4_. Because CoO is also a p‐type semiconductor, their coexistence forms a p–p heterojunction [63, 64]. This structural transformation significantly affects the electronic properties of the material, particularly changes in the bandgaps. CoO has a wider bandgap compared with Co_3_O_4_ with a slightly lower work function, and the coexistence of Co_3_O_4_ and CoO forms a heterostructure, where the alignment of the conduction and valence bands is determined by their relative electronic properties. The valence band of CoO was slightly lower than that of Co_3_O_4_, creating an energy barrier for hole transfer; furthermore, the conduction band offset facilitated electron transfer between Co_3_O_4_ and CoO. This charge transfer significantly expands the hole accumulation layer, resulting in a low base resistance in ambient air [54]. We suggest that the superior sensing performance of the sensors for H_2_S gas compared with other gases can be attributed to their aligned structure, which includes a large specific surface area, a large Co^2+^/Co^3+^ ratio, and an abundance of vacancy defects. Upon exposure to H_2_S gas, specific surface reactions release trapped electrons that effectively neutralize the accumulated holes, causing a drastic increase in the sensor resistance [65]. The reaction kinetics of the H_2_S gas sensing process in the cobalt oxide NPs can be explained by reaction Equations ((2), (3), (4), (5), (6)) [66, 67], which are summarized in the schematic in Figure 4F.

(2) $$O_{2\left(g\right)}+e^{-}\to O_{2\left(ads\right)}^{-}+h^{*}$$

(3) $$O_{2\left(ads\right)}^{-}+e^{-}\to 2O_{\left(ads\right)}^{-}+h^{*}$$

(4) $$O_{\left(ads\right)}^{-}+e^{-}\to O_{\left(ads\right)}^{2-}+h^{*}$$

(5) $$H_{2}S_{\left(g\right)}+3O^{-}+3h^{*}\to H_{2}O_{\left(g\right)}+SO_{2\left(g\right)}$$

(6) $$H_{2}S_{\left(g\right)}+3O^{2-}+6h^{*}\to H_{2}O_{\left(g\right)}+SO_{2\left(g\right)}$$

**FIGURE 5:**
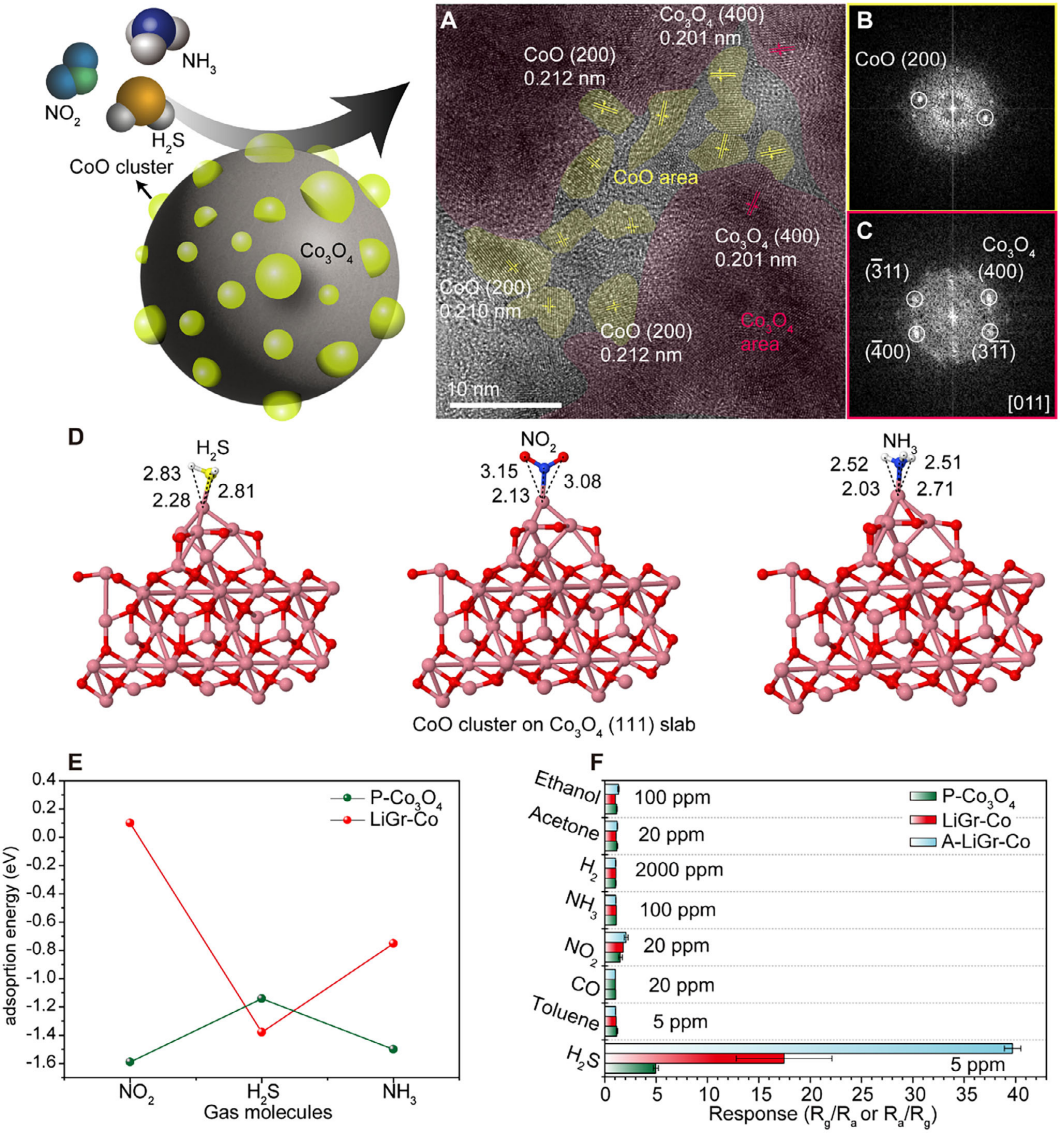
(A) High‐resolution TEM images of the LiGr‐Co NPs, (B,C) corresponding diffraction pattern of Co_3_O_4_ and CoO area via FFT. (D) Optimal stable adsorption configurations of the LiGr‐Co structure with a CoO cluster with H_2_S, NO_2_, and NH_3_. (E) Variations of calculated adsorption energy for the CoO cluster adsorbing NO_2_, H_2_S, and NH_3_ on the Co_3_O_4_ (111) slab structure. (F) Responses of gas sensors based on P‐Co_3_O_4_, LiGr‐Co, and A‐LiGr‐Co samples to various concentrations of different gases at the optimal temperature.

The LiGr process was used to ferromagnetic cobalt oxide. Under controlled current conditions, CoO clusters selectively formed on the Co_3_O_4_ surface without compromising the spinel structure. This process is outlined the HR‐TEM images, which provide evidence of the CoO clusters (marked in yellow regions) distributed on the Co_3_O_4_ nanostructures. Specifically, Figure 5A shows the lattice fringes corresponding to the CoO (200) (d‐spacing = 0.212 nm) and Co_3_O_4_ (400) planes (d‐spacing = 0.201 nm). The coexistence of these two phases confirms the partial reduction of Co_3_O_4_ into CoO clusters (Figure 5B,C). To understand the selective adsorption of H_2_S gas molecules on the oxygen‐deficient Co_3_O_4_ surfaces, we calculated the adsorption energies of H_2_S, NH_3_, and NO_2_ gases based on spin‐polarized DFT, as shown in Figure 5D. From the analysis of the adsorption properties of H_2_S, NO_2_, and NH_3_ on the CoO cluster on the Co_3_O_4_ (111) slab, the distinction factor was attributed to the charge‐transfer mechanisms of the oxidizing and reducing gases. NO_2_, an electron acceptor, withdraws charge from the surface, strengthening its N–O bonds and causing a bond contraction (1.22 Å → 1.02 Å) [68, 69]. In contrast, H_2_S and NH_3_, as electron donors, transfer charge to the surface, weakening their respective bonds and leading to H–S (1.33 Å → 1.35 Å) and N–H (1.01 Å → 1.03 Å) elongation [70, 71, 72, 73]. In other words, the stronger interaction with the H_2_S molecule was further evidenced by the highly negative adsorption energy of the H_2_S gas molecules and the superior gas sensor response of LiGr‐Co. The adsorption energies are defined as

(7) $$\Delta \;E_{\mathrm{ads}}=-E_{\frac{\mathrm{gas}}{\mathrm{slab}}}+E_{\mathrm{slab}}+E_{\mathrm{gas}}$$

as shown in Figure 5E, where *E*_gas/slab_, *E*_slab_, and *E_gas_* are the total DFT energies of the slab with the adsorbed gas, slab, and gas, respectively. From the present calculations, the CoO cluster on the (111) Co_3_O_4_ surface absorbs a H_2_S gas molecule with a stronger adsorption energy of −1.38 eV than the other two molecules. However, the adsorption energy values for P‐Co_3_O_4_ when interacting with various gas molecules—NO_2_, H_2_S, and NH_3_—indicated that P‐Co_3_O_4_ did not exhibit significant selectivity toward any particular gas. Specifically, the adsorption energy for NO_2_ is approximately −1.59 eV, for H_2_S is approximately −1.14 eV, and for NH_3_ is approximately −1.50 eV, showing relatively small variations across different gas species. The moderate and closely spaced adsorption energy values suggest that P‐Co_3_O_4_ interacts weakly and nonpreferentially with the target gases, lacking a strong and distinctive interaction that would allow it to effectively differentiate one gas from another. Figure 5F shows the gas response values of the A‐LiGr‐Co sensor to 5 ppm of H_2_S and various interfering gases, including 20 ppm NO_2_ and acetone gases, 100 ppm NH_3_, CO, toluene, and ethanol gases, and 2000 ppm H_2_ gas. Among these interference gases, the response to 20 ppm NO_2_ gas remained relatively low at 1.87, and the responses to the other reducing gases and VOCs were completely negligible, which firmly demonstrates the exceptional H_2_S gas selectivity of our A‐LiGr‐Co‐based gas sensor in complex gas environments.

## 3 Conclusion

In this work, we investigate the structural and magnetic properties of cobalt oxide NPs and their influence on H_2_S gas‐sensing performance. Co_3_O_4_, a normal spinel with Fd‐3m symmetry, features a cubic close‐packed arrangement of oxygen anions, indicating its antiferromagnetic properties. The introduction of LiGr facilitates the transformation of P‐Co_3_O_4_ into ferromagnetic cobalt oxide with few‐nanometer‐scaled particles and numerous oxygen defects. The LiGr‐Co NPs based H_2_S gas sensor exhibits significant enhancement in gas‐sensing performance owing to increased surface oxygen vacancies and a larger Co^2+^/Co^3+^ ratio. Raman spectroscopy and XRD pattern analyses confirm the presence of a pure spinel structure and the formation of microsized crystalline domains. ESR and magnetic hysteresis measurements revealed that the LiGr‐Co NPs exhibited ferromagnetic behavior, which is attributed to the uncompensated Co^2+^ ions and the high concentration of oxygen vacancies. Our findings highlight that, when subjected to a magnetic field during the spray coating process, LiGr‐Co NPs form a micropillar array that improves the efficiency of H_2_S gas adsorption and electron transfer. This alignment significantly reduces the base resistance and enhances the effectiveness of the sensing material as an H_2_S gas sensor. Notably, the aligned A‐LiGr‐Co samples exhibit superior sensitivity and selectivity for H_2_S detection at relatively low operating temperatures. DFT results demonstrate that H_2_S gas exhibits the strongest adsorption energy on the CoO cluster, indicating a significant charge‐transfer interaction, whereas NO_2_ and NH_3_ gases show weaker adsorption. This correlates well with the gas‐sensing performance, where the response to 20 ppm of NO_2_ was only 1.87, whereas the response to 5 ppm of H_2_S gas was 39.7 at 150°C. Utilizing the magnetic‐field‐induced multichannel micropillar array, suggesting that the optimized structural design significantly amplified the gas adsorption and charge‐transfer effects, improving the H_2_S gas sensor performance. Furthermore, the linear relationship between the response and the H_2_S gas concentration at higher levels is attributed to the supersaturated absorption of A‐LiGr‐Co. The calculated LOD was 195 ppb, further confirming the high efficiency of the A‐LiGr‐Co‐based H_2_S gas sensor. These results highlight the potential of LiGr‐Co NPs as highly effective materials for gas‐sensing applications, particularly for detecting H_2_S gas.

## 4 Experimental Section/Methods

### 4.1 Materials and Chemicals

Co_3_O_4_ NPs (100 nm, 99.9% purity) were obtained from Sigma‐Aldrich. Lithium foil (99.999% purity) was obtained from Honjo Chemical Corp., and copper foil was acquired from UACJ Foil Corp. Polyvinylidene fluoride (PVDF, Sigma‐Aldrich), 1‐methyl‐2‐pyrrolidinone (NMP, Merck), and Super P (TIMCAL, Alfa Aesar) were mixed in a slurry. The electrolyte used in the LiGr process was a 1 m LiPF_6_ solution dissolved in a mixture of ethyl carbonate and diethyl carbonate (EC/DEC, 1/1 v/v, EnChem). All the other reagents were procured from Sigma‐Aldrich or Merck and used without further purification.

### 4.2 Method of Lithiation‐Based Galvanostatic Reduction (LiGr) of Co_3_O_4_ NPs

The reduced Co_3_O_4_ NPs were prepared using the following procedure: The purchased P‐Co_3_O_4_, a polyvinylidene fluoride binder, and Super P were dispersed in 1‐methyl‐2‐pyrrolidinone and mixed in a slurry with a weight ratio of 90:5:5. A binder was added to structure the slurry, and Super P was included as a conductive material to ensure a sufficient reduction reaction within the electrochemical cell. This suspension was uniformly spread into a smooth layer, 200 µm thick, onto the copper foil. The slurry‐coated copper foil was then dried for 24 h in a vacuum oven at 60°C. A 2032 coin‐type cell was assembled using a Li foil as the counter electrode and 1 m LiPF_6_ in EC/DEC as the electrolyte. The cell assembly involved the Li foil, Co_3_O_4_ electrodes, and a porous membrane (Celgard) serving as the counter/reference electrode, working electrode, and separator, respectively, with the electrolyte in an Ar‐filled glove box (O_2 _< 0.1 ppm and H_2_O < 0.1 ppm). The LiGr process was monitored using a battery cycler (WBCS3000Le32, Won‐A‐Tech) to maintain a constant current density of 100 µA cm^−2^. The discharge voltage was controlled at 1 V versus Li/Li^+^. After the process, the resulting samples were washed several times with acetone, deionized (DI) water, and a 1 × 10^−3^ m acetic acid solution to remove the residual electrolytes, lithium oxide, and impurities. The solution obtained after washing was heat‐treated in an oven at 60°C for 12 h, resulting in a powder known as LiGr‐Co.

### 4.3 Characterization

Ex situ field‐emission SEM (FE‐SEM; SUPRA55VP, Carl Zeiss), transmission electron microscopy (TEM, JEM‐2100F), and Cs‐corrected scanning transmission electron microscopy (STEM, ARM200F) were employed to observe the atomic structures. XRD (SmartLab 9 kW, Rigaku) with Cu Kα radiation (*λ* = 1.5406 Å) was used to identify the phase transitions. X‐ray photoelectron spectroscopy (XPS, Cu Kα, Thermo Fisher Scientific Co.) was utilized to obtain the spectra of Co and O chemical bonds. Raman spectroscopy (optical/Raman microscope, Olympus) was used to examine the chemical state of the Co_3_O_4_ surface. The surface morphology of the alumina substrate was examined using a ContourX‐1000 3D optical profilometer (Bruker Daltonics Inc., Billerica, MA, USA). The magnetic properties were analyzed by using a superconducting quantum interference device (SQUID, Quantum Design Inc.) and an electron spin resonance (ESR5000, Bruker Daltonics Inc.)

*Fabrication of spray‐coated aligned A‐LiGr‐Co*: To systematically evaluate the gas sensing properties, the sensor device was fabricated on an alumina substrate with dimensions of 1.5 cm × 1.5 cm. Interdigitated electrodes were patterned onto the substrate surface to measure the electrical resistance changes. The electrode fabrication process was carefully conducted by sequentially depositing a 10 nm thick Cr adhesion layer using an electron‐beam evaporator and a 100 nm thick Au conductive layer using a thermal evaporator. As depicted in the schematic measurement setup, the active interdigitated area possesses a gap distance of electrode pad with 2 mm, a finger width of 0.25 mm, and a gap distance of 0.25 mm. Then, the synthesized A‐LiGr‐Co sensing material was uniformly coated onto these highly stable interdigitated electrodes. Subsequently, suspensions of 2 mg mL^−1^ of P‐Co_3_O_4_, LiGr‐Co, and A‐LiGr‐Co with DI water were dropped onto the interdigital Au electrode gap under a 0.8 T magnetic field using a neodymium magnet positioned perpendicular to the electrodes. The interdigital electrodes, with a 10 µm spacing, were fabricated using standard microfabrication procedures. During an evaporation process for 6 h in an oven at 100°C, A‐LiGr‐Co aligned controllably between the interdigital electrodes, forming a uniform aligned H_2_S gas sensor.

*Gas‐sensing tests*: The fabricated gas sensors were placed inside a gas chamber within a box furnace under temperature control (Figure S5). The properties of these sensors were evaluated at various temperatures. Analyte gases, including hydrogen sulfide (H_2_S), nitrogen dioxide (NO_2_), ammonia (NH_3_), hydrogen (H_2_), toluene (C_6_H_5_CH_3_), and acetone (CH_3_COCH_3_), were individually stored in gas tanks at concentrations ranging from 20 to 100 ppm (Seoul Specialty Gases and JC Gas, South Korea) and subsequently introduced into the sensor chamber for evaluating the gas‐sensing performance. The chamber was equipped with a gas inlet pipe connected to a mass flow controller, which regulated the test gas concentration by delivering a proportional volume through a controlled injection system. Prior to and after each target gas exposure, dry air (comprising 78% N_2_ and 21% O_2_) was purged into the chamber to stabilize the baseline. Once the sensor resistance was stabilized in ambient air, the analyte gas was introduced via the inlet system. To assess repeatability and optimize response behavior, the sensors were exposed to H_2_S gas for at least 15 cycles. The sensor responses were recorded for each cycle, and the gas response values were calculated after reaching steady state. The flow rate was set to 500 standard cubic centimeters per minute (sccm) using mass flow controllers, with air as the background gas. The sensor resistances in air (*R*_a_) and in the presence of the target gas (*R*_g_) were measured, and the sensor responses were defined as *R* = *R*_g_/*R*_a_ for H_2_S gas as reducing gases and *R* = *R*_a_/*R*_g_ for oxidizing gases. To investigate the effects of humidity, H_2_S gas measurements were conducted in both dry and humid air (relative humidity (RH) 0%–90%, measured at 25°C). The air humidity was controlled by adjusting the rate of the mass flow controller connected to the bubbling system. Immediately before loading into the gas chamber, the actual temperature and RH of the humid air were measured using an RH probe (HF5 transmitter; Rotronic, USA).

### 4.4 Density Functional Theory Calculations

The calculations were performed using the Vienna Ab initio Simulation Package (VASP) [74, 75, 76], which uses a plane‐wave basis set for the valence electron states. The projector augmented wave (PAW) scheme and generalized gradient approximation (GGA) with the exchange‐correlation functional of Perdew, Burke, and Ernzerhof (PBE) were used [77]. As PBE does not account for van der Waals (vdW) forces, the DFT‐D3 method with the Becke–Johnson damping function was used [78, 79]. In addition, to correct for the large self‐interaction error of the 3d electrons of Co, a Hubbard‐like repulsion term was added using the approach of Dudarev (DFT+U) [80], with *U*_eff_  =  *U*  −  *J*  =  4.5 eV.

A plane‐wave cutoff energy of 700 eV was used for the structural energy calculations. For a (2  ×  2) slab (*a*  =  *b*  =  11.4,  γ  =  120°), the Brillouin zone was sampled using a 3 × 3 × 1 Monkhorst–Pack mesh. In the calculations, periodic boundary conditions with a 4‐CoO cluster on the stoichiometric 16(Co_3_O_4_) (111) pristine surface were employed in all three directions with a vacuum gap of at least 15 Å in the *z*‐direction to separate the two distinct surfaces. All atoms were relaxed using the conjugate gradient method until the residual forces on constituent atoms became smaller than $1\times 10^{-2}\;\mathrm{eV}/Å$. We carefully checked the convergence of the atomic configurations and relative energies with respect to the plane‐wave energy cutoff, supercell size, and k‐point mesh size.

## Conflicts of Interest

The authors declare no conflict of interest.

## Supporting information




**Supporting File**: smll73475‐sup‐0001‐SuppMat.docx.

## Data Availability

Data will be made available on reasonable request.
