# Supplementary material for: Ferromagnetic Cobalt Oxide With Structural Distortion and Oxidation State Changes for Hydrogen Sulfide Gas Detection
Source: Small. 2026 Apr 21;22(32):e73475. doi: 10.1002/smll.73475 (PMC13244406; doi:10.1002/smll.73475)
Supplement: Supplementary file 1 — Supporting File: smll73475‐sup‐0001‐SuppMat.docx. [file SMLL-22-e73475-s001.docx]

Supporting Information

Ferromagnetic Cobalt Oxide with Structural Distortion and Oxidation State Changes for Hydrogen Sulfide Gas Detection

Shin Joon Kang, Chang Yoon Kim, Min Chan Kim, Sunhyeong Kwon, Joon-Shik Park*, and Hyung Mo Jeong*

*
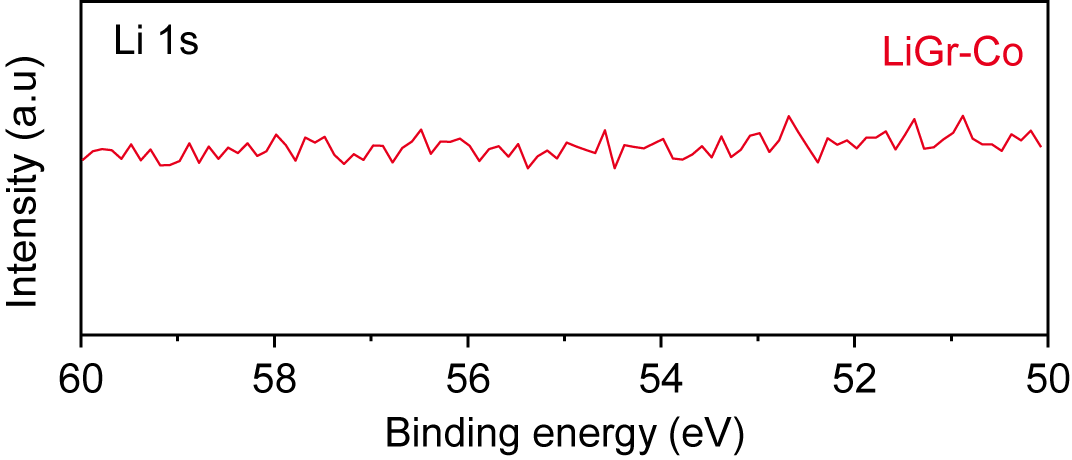
*

**Figure S1.** Li 1s XPS spectra of LiGr-Co particles after LiGr process.


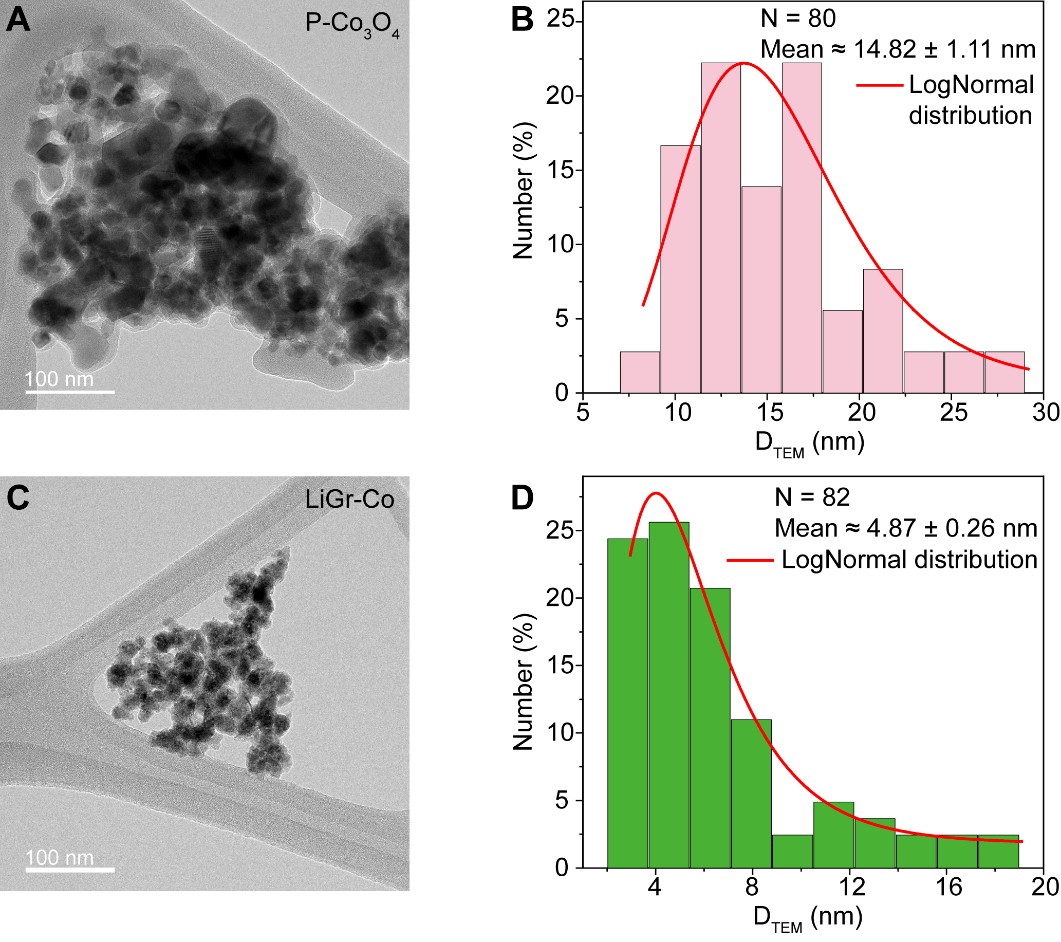


**Figure S2.** (A,C) TEM images with low magnification and particle size distribution histograms of (B) P-Co_3_O_4_ and (D) LiGr-Co.


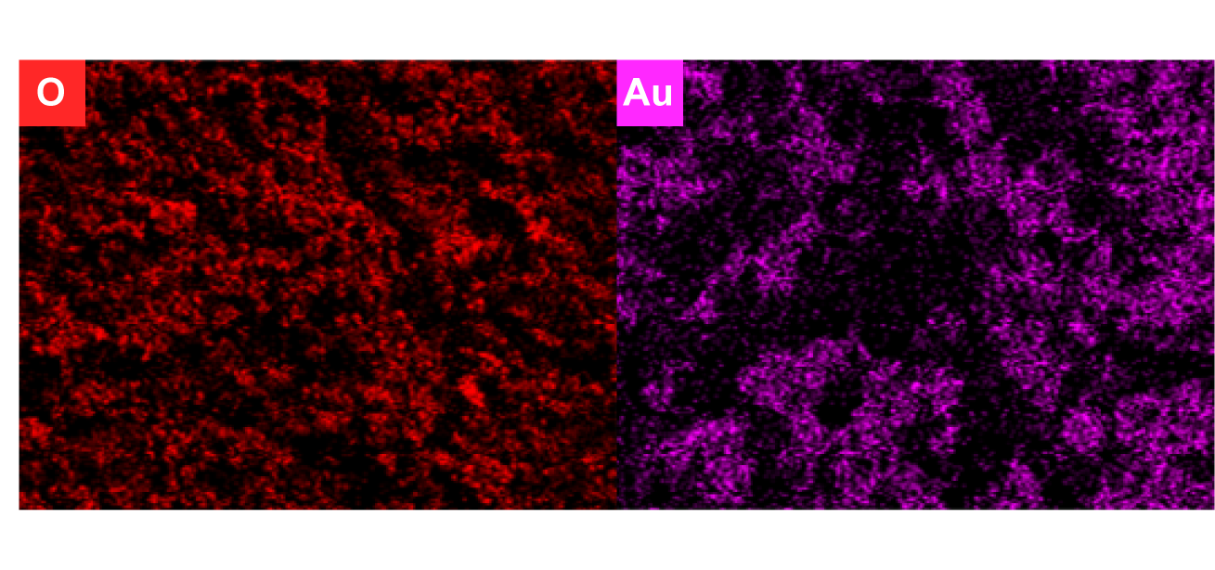


**Figure S3.** O and Au elemental EDS mapping of SEM image of P-Co_3_O_4_ sensor.


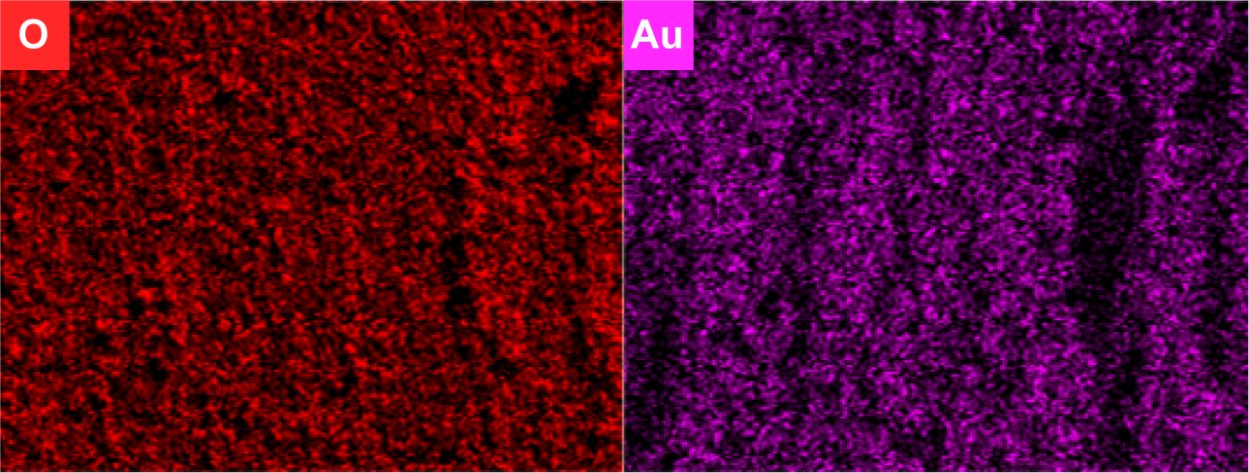


**Figure S4.** O and Au elemental EDS mapping of SEM image of A-LiGr-Co sensor.


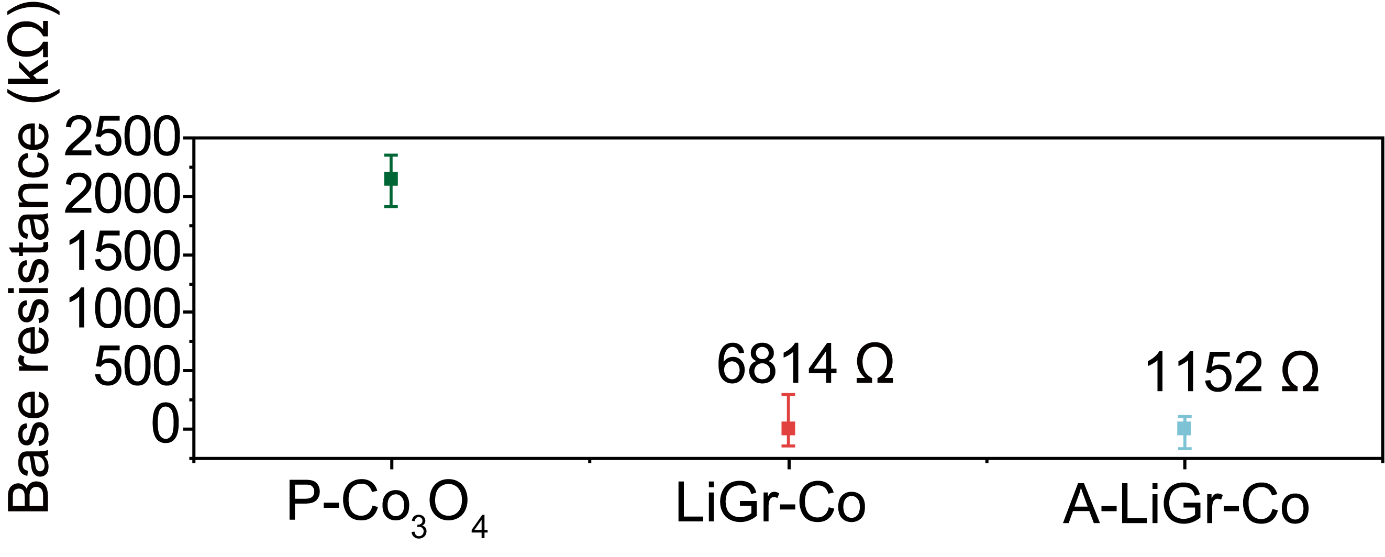
**Figure S5.** Base resistance values for P-Co_3_O_4_, LiGr-Co and A-LiGr-Co at the 200°C. Error bars represent the standard deviation of the measurements.


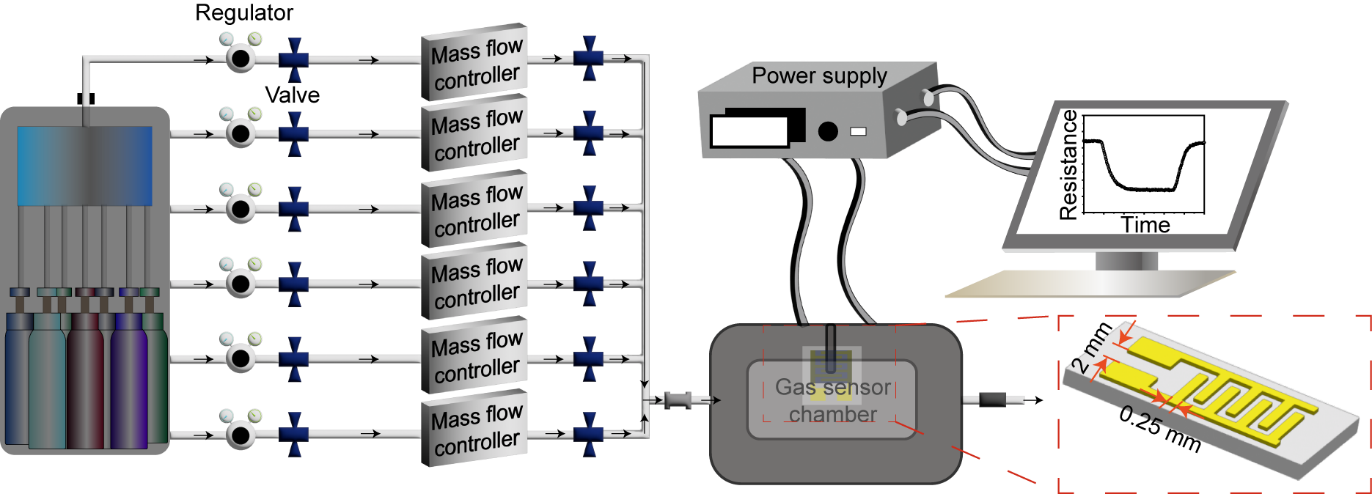
**Figure S6.** Schematic illustration of the overall gas sensing system and device configuration.


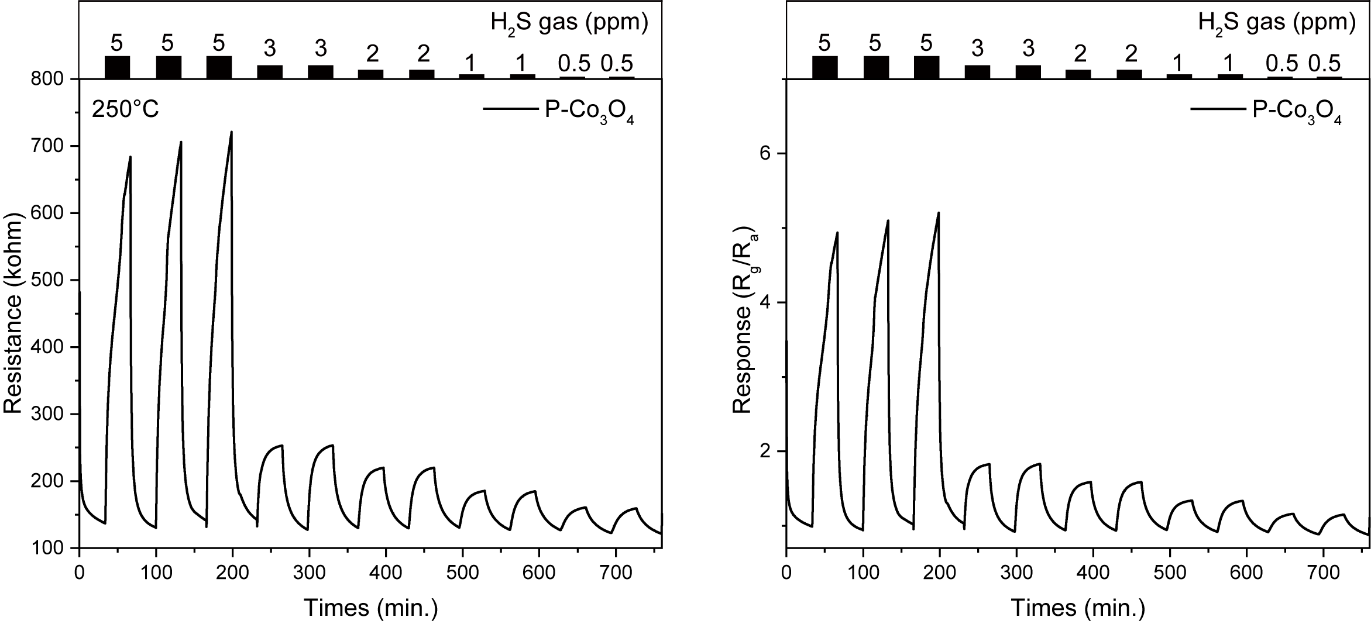
**Figure S7.** Resistance variation and corresponding sensor response of P-Co_3_O_4_ sensor under various concentrations of H_2_S gas.


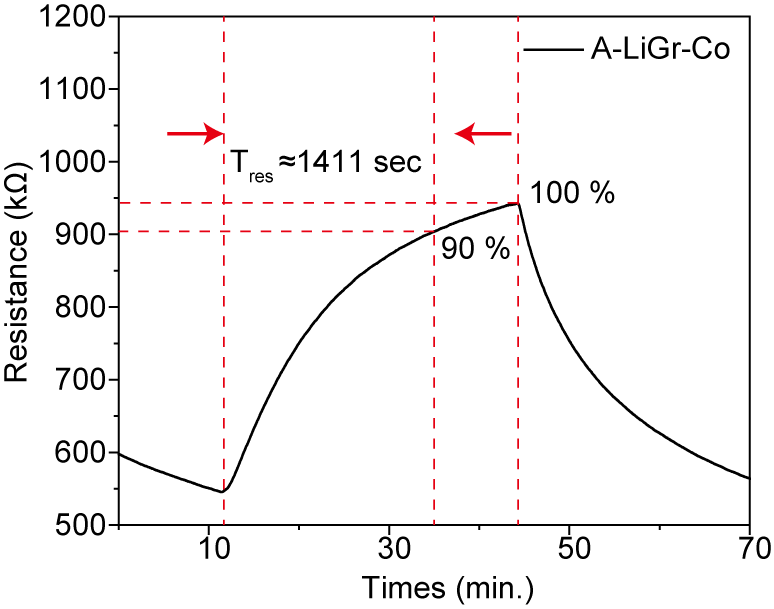
**Figure S8.** Calculated response time of A-LiGr-Co gas sensor to 1 ppm H_2_S gas.


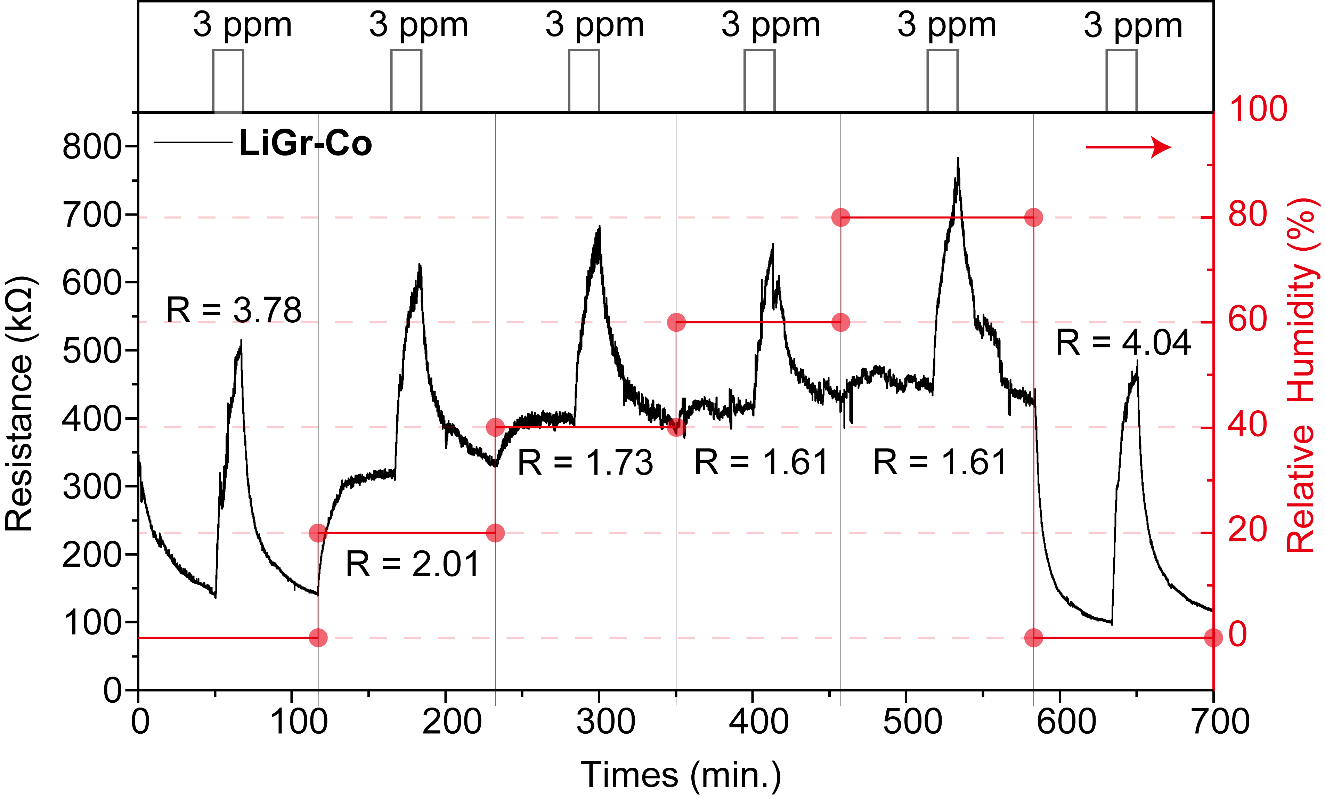


**Figure S9.** Sensing performance of the LiGr-Co sensor toward 3 ppm H_2_S gas under varying humidity conditions at an operating temperature of 150 °C.
